# Acidified drinking water improves motor function, prevents tremors and changes disease trajectory in *Cln2*^*R207X*^ mice, a model of late infantile Batten disease

**DOI:** 10.1038/s41598-023-46283-w

**Published:** 2023-11-06

**Authors:** Attila D. Kovács, Jose L. Gonzalez Hernandez, David A. Pearce

**Affiliations:** 1 Pediatrics and Rare Diseases Group, Sanford Research, 2301 E. 60th Street N., Sioux Falls, SD 57104 USA; 2 Department of Pediatrics, Sanford School of Medicine, University of South Dakota, Sioux Falls, SD USA; 3 Department of Agronomy, Horticulture, and Plant Science, South Dakota State University, Brookings, SD USA; 4 Department of Biology and Microbiology, South Dakota State University, Brookings, SD USA

**Keywords:** Microbiology, Neuroscience

## Abstract

Batten disease is a group of mostly pediatric neurodegenerative lysosomal storage disorders caused by mutations in the CLN1–14 genes. We have recently shown that acidified drinking water attenuated neuropathological changes and improved motor function in the *Cln1*^*R151X*^ and *Cln3*^*−/−*^ mouse models of infantile CLN1 and juvenile CLN3 diseases. Here we tested if acidified drinking water has beneficial effects in *Cln2*^*R207X*^ mice, a nonsense mutant model of late infantile CLN2 disease. *Cln2*^*R207X*^ mice have motor deficits, muscle weakness, develop tremors, and die prematurely between 4 and 6 months of age. Acidified water administered to *Cln2*^*R207X*^ male mice from postnatal day 21 significantly improved motor function, restored muscle strength and prevented tremors as measured at 3 months of age. Acidified drinking water also changed disease trajectory, slightly delaying the death of *Cln2*^*R207X*^ males and females. The gut microbiota compositions of *Cln2*^*R207X*^ and wild-type male mice were markedly different and acidified drinking water significantly altered the gut microbiota of *Cln2*^*R207X*^ mice. This suggests that gut bacteria might contribute to the beneficial effects of acidified drinking water. Our study demonstrates that drinking water is a major environmental factor that can alter disease phenotypes and disease progression in rodent disease models.

## Introduction

Late infantile CLN2 disease is a recessively inherited, progressive neurodegenerative disorder that belongs to neuronal ceroid lipofuscinoses (also known as Batten disease), a group of lysosomal storage diseases with ceroid lipofuscin as storage material. CLN2 disease is caused by mutations in the *CLN2* gene encoding the lysosomal protease, tripeptidyl peptidase 1 (TPP1)^1^. The disease begins at 2–4 years of age with seizures and ataxia followed by visual impairment and progressive cognitive and motor dysfunction, leading to death by 6–15 years of age^2^. It is still unknown how TPP1 deficiency results in brain atrophy. Since 2017, enzyme replacement therapy by intracerebroventricular infusion has been available for patients with CLN2 disease. This treatment with a recombinant TPP1 (cerliponase alfa by BioMarin Pharmaceutical Inc.) slows the decline of motor and language functions in symptomatic CLN2 disease patients 3 years of age and older^3, 4^.

We have recently generated a novel mouse model of the disease, *Cln2*^*R207X*^ mice. The *Cln2* mutation in these mice corresponds to the most frequent disease-causing human nonsense mutation. In *Cln2*^*R207X*^ mice, due to the nonsense mutation that generates a premature stop codon, the mutant *Cln2* mRNA level is markedly decreased by nonsense-mediated mRNA decay. Accordingly, TPP1 enzyme activity is dramatically reduced in the organs of *Cln2*^*R207X*^ mice. Less than 1% of the wild-type TPP1 activity was measured in the liver, kidney, cerebral cortex and cerebellum of *Cln2*^*R207X*^ mice^5^. *Cln2*^*R207X*^ mice have a progressive disease course with significant neurological abnormalities, tremors, and neuropathological changes, such as accumulation of lysosomal storage material and astrocytic activation in the brain at 3 months of age. *Cln2*^*R207X*^ mice prematurely die between 4 and 6 months of age^5^.

The type of drinking water (tap, distilled, autoclaved, acidified, neutral, alkaline) is a major environmental factor that can affect the physiology of experimental animals, but it is rarely reported in scientific publications. At numerous animal facilities, acidification of drinking water with HCl is used to eliminate pathogenic bacteria. We have recently shown in the *Cln3*^*−/−*^ and *Cln1*^*R151X*^ mouse models of juvenile CLN3 and infantile CLN1 diseases that acidified drinking water attenuated neuropathological changes in a disease-specific manner and provided disease-dependent beneficial effects on neurological function^6, 7^. In both *Cln3*^*−/−*^ and *Cln1*^*R151X*^ mice, the effects of acidified drinking water were accompanied by significant changes in the gut microbiota composition^6, 7^.

In the current study, we tested if acidified drinking water provides disease-modifying effects in the *Cln2*^*R207X*^ nonsense mutant mouse model of late infantile CLN2 disease. The effect of acidified drinking water on the altered gut microbiota composition of *Cln2*^*R207X*^ mice was also examined.

## Materials and methods

### Animals

We maintained *Cln2*^*R207X*^ mice on a mixed 129S6/SvEv;C57BL/6J genetic background and 129S6/SvEv;C57BL/6J wild-type (WT) mice in our mouse colony. *Cln2*^*R207X*^ mice and WT mice were not littermates; in our colony, *Cln2*^*R207X*^ and WT mice were separately maintained. Mice were housed in ventilated microisolator cages (4–5 mice/cage), with ad libitum food (Teklad Global 2918 diet; Harlan Laboratories, Indianapolis, IN) and water (non-acidified tap water, pH 8.4), with a 14-h light, 10-h dark cycle. *Cln2*^*R207X*^ and WT mice were randomly assigned to receive acidified drinking water from 21 days of age (weaning). To acidify tap water with HCl (pH 2.5–2.9; average pH: 2.8) a Technilab BMI BV water acidification system (Tecniplast USA, West Chester, PA) was used. In the behavioral and gut microbiota experiments, only male mice were used. In a comparative study, we previously demonstrated that male mouse models of juvenile CLN3 disease (*Cln3*^*−/−*^ and *Cln3*^*Δex7/8*^ male mice) display more pronounced disease phenotypes than females and therefore, are more appropriate for testing new therapies^8^. Consequently, since we made the *Cln2*^*R207X*^ mouse model to test novel therapies, when *Cln2*^*R207X*^ mice were characterized, only the neurological phenotypes of male mice were determined^5^.

To determine the survival curves, both males and female mice were used.

*Cln2*^*R207X*^ mice have a severe disease phenotype, they start trembling around 90 days of age and die within a few months. Therefore, from 90 days of age, *Cln2*^*R207X*^ mice were monitored daily and were euthanized when morbidity criteria (i.e., immobility, difficulties in feeding) were observed. Mice were euthanized by carbon dioxide inhalation in accordance with the AVMA Guidelines for the Euthanasia of Animals: 2020 Edition.

All animal procedures were approved by the Sanford Research Animal Care and Use Committee and were in compliance with NIH policies and the guidelines of the Animal Welfare Act. To report the animal experiments we followed the recommendations in the ARRIVE guidelines^9^.

### Behavioral testing

Behavioral testing was carried out during the light phase. In the behavioral testing room, the lights were dimmed. Before starting the behavioral tests, the mice were weighed and let to adapt to the room for at least 20 min. The modified vertical pole test was the first test, followed by the wire hanging test and the force-plate actimeter.

The behavioral test results and the weight data were analyzed by 2-way ANOVA with Tukey’s post-test for multiple comparisons in GraphPad Prism 7.04 (GraphPad Software, San Diego, CA).

#### Modified vertical pole test

A modified version of the vertical pole test was used to assess motor function as we previously described^6–8^. The test started by putting the mouse at the top of the pole, head downward, and the time the mouse took to climb down the pole was measured in 5 consecutive trials. Following the climb down trials, the same mouse was put at the top of the pole, head upward, and the time the mouse took to turn completely downward was measured in 4 consecutive trials. Each test trial lasted a maximum of 60 s (to avoid fatigue). When a mouse fell, a 60 s score was recorded.

#### Wire hanging test

To test muscle strength, mice were placed in the center of a stainless-steel cooling rack for baking (20.32 cm × 29.85 cm; grid spacing: 1.27 cm square). The rack was inverted and held 46 cm above a cage containing bedding. The time mice could remain suspended was measured; 60 s was the cut-off time to prevent exhaustion. Each mouse was tested 5 consecutive times and the average time of staying suspended (latency to fall) was calculated.

#### Force-plate actimeter

The force-plate actimeter (BASi, West Lafayette, IN, USA) monitors several behavioral parameters in a freely moving mouse, such as locomotor activity (e.g., covered area, overall distance of movements, number and total degrees of turns, and distribution of the animal’s activity across the plate called spatial statistic), focused stereotypy score (number of occasions when the mouse is rearing, scratching, grooming or head bobbing), number of times during the test when a bout of low mobility occurred, and tremors over different frequency ranges can be measured^5^. Mice were tested in the force-plate actimeter as we previously described^6^ with some modification. Briefly, mice were put in the force-plate actimeter and the above-described behavioral parameters were recorded for 10.24 min. The recorded parameters were analyzed using FPAAnalysis software version 1.10.01 (BASi, West Lafayette, IN, USA; https://www.basinc.com/assets/library/manuals/FPA.pdf).

### Analysis of the gut microbiota

When each mouse finished the modified vertical pole test, fecal pellets were aseptically collected from the pole base in a sterile 1.5-ml tube, and the tube was immediately put on dry ice. The collected fecal pellets (from 6 mice per experimental group) were stored at − 80 °C until they were shipped to MR DNA (www.mrdnalab.com, Shallowater, TX, USA) for DNA isolation and sequencing the V4 region of the bacterial 16S rRNA gene. DNA was isolated from the fecal samples using the Qiagen QIAamp DNA Stool Mini Kit (Qiagen, Valencia, CA). Sequencing was performed on the Illumina MiSeq platform with the adaptation of 16S rDNA bacterial tag-encoded FLX amplicon pyrosequencing. The sequence data were processed according to the method previously published^6^.

The sequence data provided by MR DNA underwent bioinformatic and statistical analyses using the Microbial Genomics Module in CLC Genomics Workbench vs 21.0.3 (Qiagen). Following quality trimming (Q = 20 and adapter trimming) and exclusion of chimeric reads, the obtained operational taxonomic units (OTUs) were aligned to the SILVA v132 database^10^ at 97% similarity. The Chao-1 bias-corrected index was used to measure alpha diversities^11, 12^. To determine the statistical significance for differences in alpha diversity, a Kruskal–Wallis test with Mann–Whitney U pairwise comparison was used. The Jaccard dissimilarity index was used for Principal Coordinate Analysis (PCoA) to compare beta diversities^13, 14^. To determine the statistical significance of differences in beta diversity between the experimental groups, a PERMANOVA analysis was applied. For pair-wise comparison of abundance at the different taxonomical levels, False Discovery Rate (FDR)-corrected p-values were calculated using a Wald test.

## Results

### Acidified drinking water in *Cln2*^*R207X*^ mice improves motor function, prevents tremors and slightly delays death

**Figure 1:**
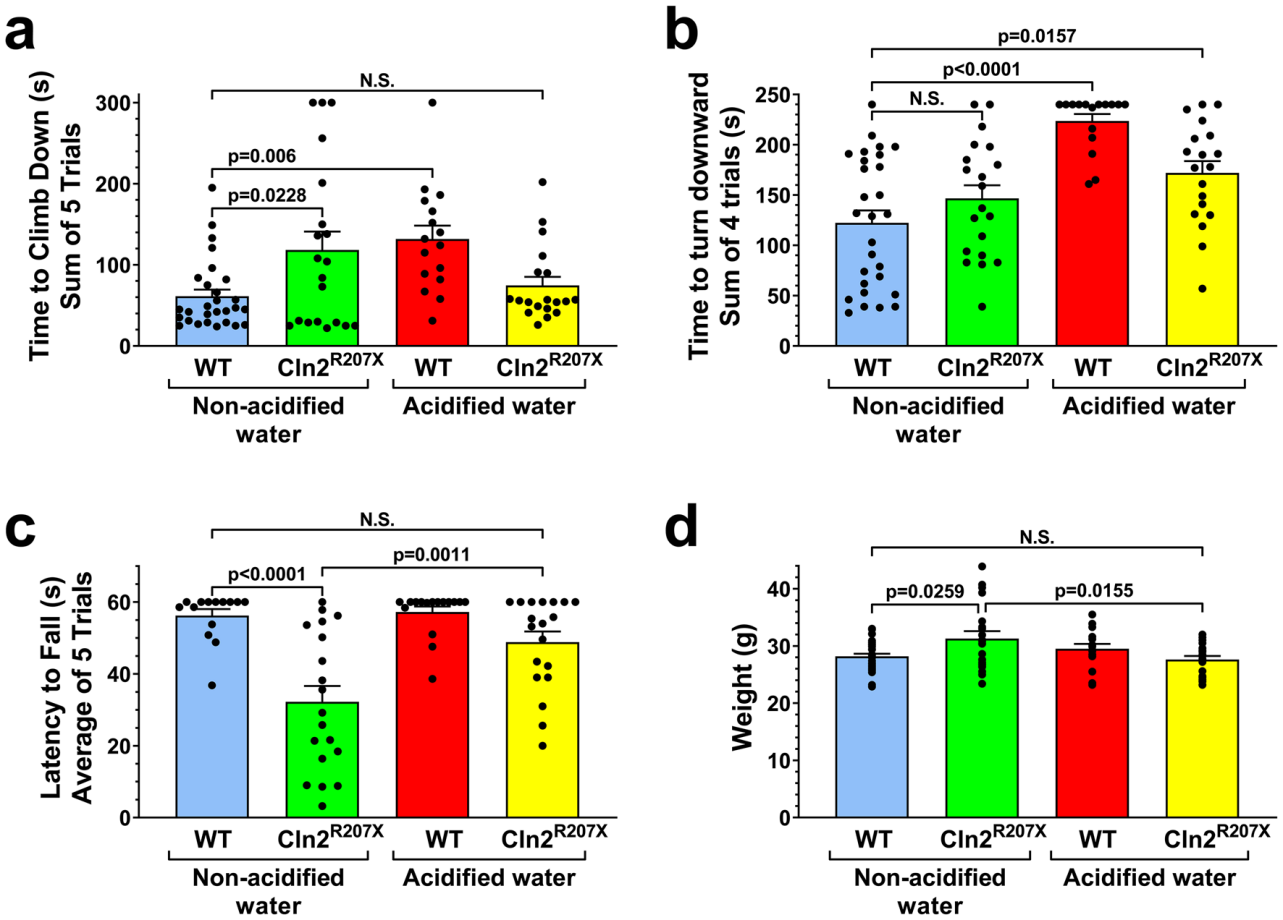
Acidified drinking water in *Cln2*^*R207X*^ mice improves motor function. *Cln2*^*R207X*^ and WT male mice either received non-acidified water or were given acidified drinking water from postnatal day 21 (weaning). At 3 months of age, the motor skills of mice were tested in a modified vertical pole test: time to climb down (**a**) and time to turn downward (**b**). Muscle strength was assessed in a wire hanging test (**c**). (**d**) Weight of the mice at 3 months. Acidified drinking water improved the ability of *Cln2*^*R207X*^ mice to climb down the pole, their performance became similar to WT mice receiving non-acidified drinking water (**a**). Acidified drinking water restored the muscle strength of *Cln2*^*R207X*^ mice to the WT level, as measured in a wire hanging test (**c**). In agreement with our previous results ^7, 15^, acidified drinking water compromised the performance of WT mice in the vertical pole test: they climbed down and turned downward significantly slower than WT mice receiving non-acidified water (**a,b**). Columns and bars represent mean + SEM and the symbols show the individual data (WT and *Cln2*^*R207X*^ on non-acidified water: 28 and 20 mice in the modified vertical pole test, and 14 and 19 mice in the wire hanging test; WT and *Cln2*^*R207X*^ on acidified water: 16 and 19 mice in both tests). Statistical significance was determined by 2-way ANOVA with Tukey’s post-test for multiple comparisons. *N.S.* not significant.

*Cln2*^*R207X*^ mice have impaired motor function, muscle weakness, develop tremors, and die prematurely between 4 and 6 months of age^5^. To investigate the potential disease-modifying effects of acidified drinking water (average pH: 2.8), *Cln2*^*R207X*^ and wild-type (WT) mice received acidified drinking water starting at weaning (postnatal day 21), and their motor function, muscle strength and tremors were evaluated at 3 months of age. In a modified vertical pole test, acidified drinking water improved the ability of *Cln2*^*R207X*^ mice to climb down the pole; their performance became similar to WT mice receiving non-acidified drinking water (Fig. 1a). Another parameter measured in the vertical pole test, the time to turn downward at the top of the pole was not improved by acidified water (Fig. 1b). In the modified vertical pole test, the climbing down phase measures agility and motor coordination, whereas the turning downward task assesses spatial orientation and motor coordination. Accordingly, acidified drinking water improved the agility and motor coordination of *Cln2*^*R207X*^ mice (Fig. 1a).

**Figure 2:**
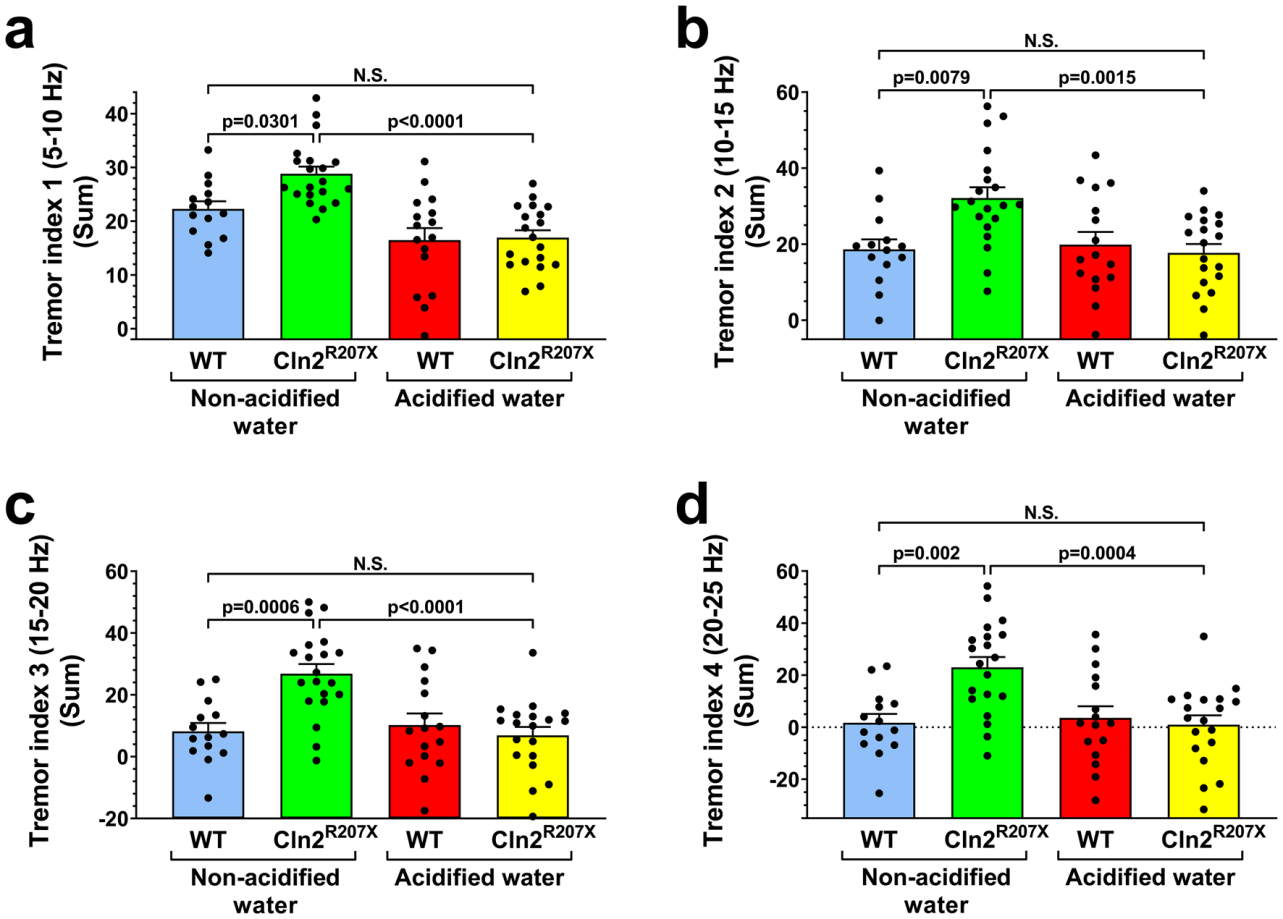
Acidified drinking water in *Cln2*^*R207X*^ mice prevents tremors. *Cln2*^*R207X*^ and WT male mice either were kept on non-acidified water or received acidified drinking water from postnatal day 21 (weaning). At 3 months of age, tremors were measured in a force-plate actimeter. Tremors were quantified as force variations at different frequencies (tremor indices 1–4) (**a–d**). *Cln2*^*R207X*^ mice on non-acidified water had intense tremors in every frequency range, and acidified drinking water prevented the development of these tremors. Columns and bars represent mean + SEM and the symbols show the individual data (WT and *Cln2*^*R207X*^ on non-acidified water: 14 and 20 mice, respectively; WT and *Cln2*^*R207X*^ on acidified water: 16 and 19 mice, respectively). Statistical significance was determined by 2-way ANOVA with Tukey’s post-test for multiple comparisons. *N.S.* not significant.

As measured in a wire hanging test, acidified drinking water restored the muscle strength of *Cln2*^*R207X*^ mice to the WT level (Fig. 1c). Acidified drinking water also prevented the development of tremors in different frequency ranges (Fig. 2). Only *Cln2*^*R207X*^ mice receiving non-acidified water displayed tremors.

In line with our previous findings^7, 15^, acidified drinking water impaired the performance of WT mice in the vertical pole test: they climbed down and turned downward significantly slower than WT mice receiving non-acidified water (Fig. 1a,b), indicating an impairment in agility, motor coordination and spatial orientation. The differential effects of acidified drinking water on the motor function in *Cln2*^*R207X*^ disease model and healthy WT mice is not surprising. While medications or treatments, especially those affecting the nervous system, have therapeutic results in disease states, they can cause adverse effects in healthy subjects.

Acidified water did not affect the muscle strength of WT mice (Fig. 1c). On non-acidified water, *Cln2*^*R207X*^ mice were significantly heavier than WT mice, indicating an abnormal weight gain (Fig. 1d). Acidified drinking water in *Cln2*^*R207X*^ mice prevented this abnormal weight gain, the weight of WT mice and *Cln2*^*R207X*^ mice receiving acidified drinking water was very similar. The decreased weight by acidified drinking water in *Cln2*^*R207X*^ mice, making them less fat and fitter, might contribute to the improved pole climbing and muscle strength. Correlation analysis, however, showed no correlation between the weight and pole climbing time in *Cln2*^*R207X*^ mice receiving non-acidified or acidified drinking water, indicating that weight was not a contributing factor to the improvement in pole climbing. While the weight of *Cln2*^*R207X*^ mice receiving acidified water did not correlate with muscle strength in the wire hanging test (p = 0.0671), the weight of *Cln2*^*R207X*^ mice receiving non-acidified water showed a negative correlation with muscle strength (heavier mice fell sooner, p = 0.0008). This indicates that the decreased weight was a contributing factor to the increased muscle strength of *Cln2*^*R207X*^ mice receiving acidified drinking water.

We also examined if acidified drinking water affected several locomotor and behavioral parameters using the force-plate actimeter (BASi, West Lafayette, IN). On non-acidified water, among all the parameters examined, only the distance traveled was significantly increased in *Cln2*^*R207X*^ mice as compared to WT mice (Fig. S1). While acidified drinking water in *Cln2*^*R207X*^ mice did not affect the distance traveled, focused stereotypies (head bobbing, grooming, rearing, scratching, etc.), or left and right turn counts, it changed the area covered, spatial statistic (space utilization), bout of low mobility and total degrees of left and right turns in comparison to WT mice receiving acidified water (Figs. S1, S2).

**Figure 3:**
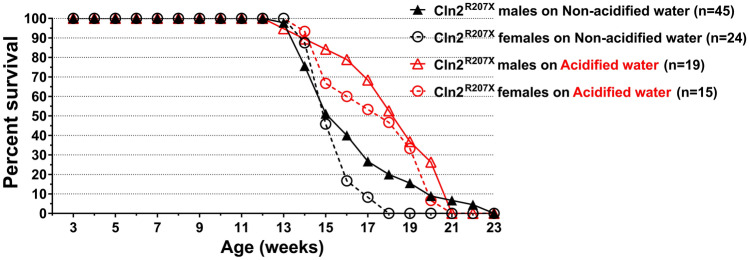
Acidified drinking water changes disease trajectory, delaying the death of *Cln2*^*R207X*^ mice. Survival curves of *Cln2*^*R207X*^ male and female mice that either were kept on non-acidified water or received acidified drinking water from postnatal day 21 (weaning). The curves represent the survival of 45 males and 24 females that received non-acidified drinking water, and of 19 males and 15 females receiving acidified drinking water from weaning.

*Cln2*^*R207X*^ mice die prematurely. Although, we did not examine neurological phenotypes in *Cln2*^*R207X*^ female mice, the death of both male and female mice was of course recorded in our *Cln2*^*R207X*^ colony, and we knew that *Cln2*^*R207X*^ females also die prematurely around the same age as *Cln2*^*R207X*^ males. Therefore, we tested the effect of acidified drinking water on the lifespan of both male and female *Cln2*^*R207X*^ mice. The median survival ages of male and female *Cln2*^*R207X*^ mice receiving non-acidified water were 15 and 16 weeks, respectively. Acidified drinking water changed disease trajectory, delaying the death of *Cln2*^*R207X*^ females and males by 3 weeks [females: p = 0.0011 by Log-rank (Mantel-Cox) test and p = 0.0152 by Gehan–Breslow–Wilcoxon test; males: p = 0.0487 by Log-rank (Mantel-Cox) test and p = 0.0091 by Gehan–Breslow–Wilcoxon test] (Fig. 3). The ratios of median survivals and their 95% confidence intervals were 1.188 (0.695–2.030) for males on acidified/males on non-acidified, and 1.200 (0.629–2.287) for females on acidified/females on non-acidified.

### Acidified drinking water significantly changes the gut microbiota composition of *Cln2*^*R207X*^ mice

**Figure 4:**
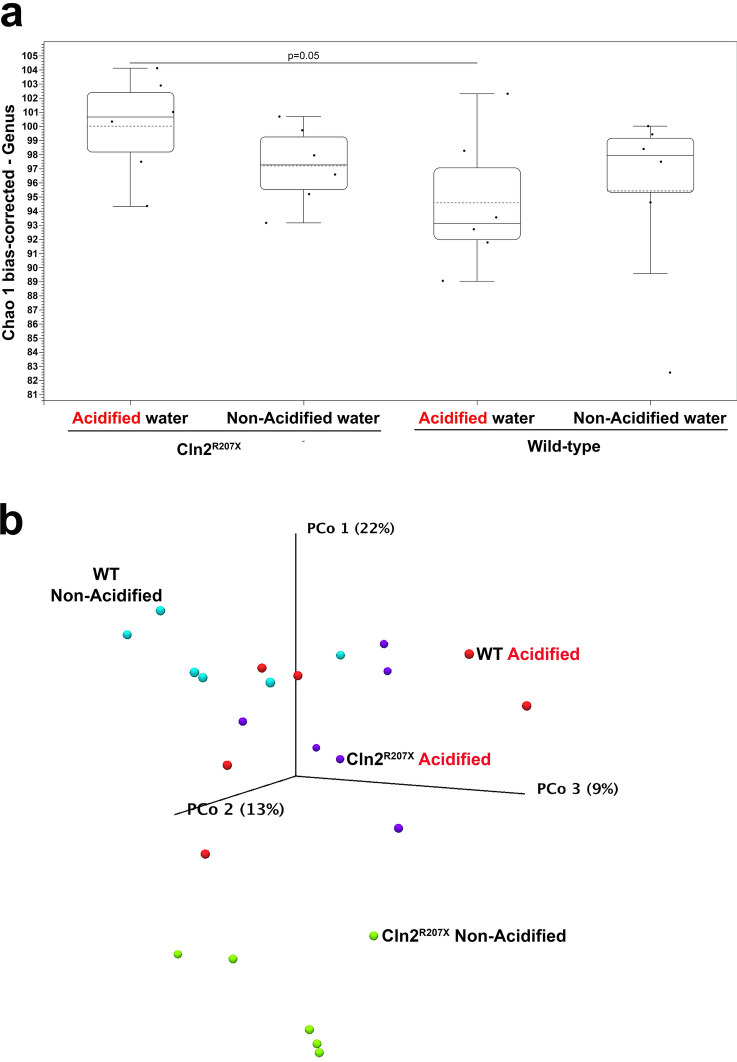
*Cln2*^*R207X*^ and WT mice have greatly different gut microbiota, and acidified drinking water causes significant alteration in the global gut microbiota composition of *Cln2*^*R207X*^ mice only. A group of *Cln2*^*R207X*^ and WT male mice was given acidified drinking water from 21 days of age (weaning). Another group of *Cln2*^*R207X*^ and WT male mice were kept on non-acidified drinking water. At 3 months of age, fecal pellets were collected for the analysis of the gut microbiota via 16S rRNA gene sequencing. (**a**) Alpha diversity, the microbial diversity within a sample, was quantified using the Chao 1 bias-corrected diversity index. Alpha diversity was similar in *Cln2*^*R207X*^ and WT mice receiving non-acidified drinking water, and acidified water did not cause statistically significant changes in either genotype. Alpha diversities in *Cln2*^*R207X*^ and WT mice receiving acidified drinking water, however, were close to be different with a p-value of 0.05. Box and whisker plot: each black dot represents a mouse (n = 6 mice). To determine the statistical significance for differences in alpha diversity, a Kruskal–Wallis test with Mann–Whitney U pairwise comparison was used. (**b**) The global gut microbiota compositions in the different groups (beta diversity) were compared by principal coordinate (PCo) analysis. Beta diversity in *Cln2*^*R207X*^ mice was significantly different from that in WT mice on both types of drinking water (p = 0.00216 and 0.02814). While acidified drinking water did not change the bacterial community structure in WT mice (p = 0.17749), it markedly altered the global microbiota composition in *Cln2*^*R207X*^ mice (p = 0.01082). Each symbol represents an individual mouse (n = 6 mice in each group). A PERMANOVA analysis was used to determine the statistical significance in beta diversity (Bray–Curtis).

Accumulated evidence indicates that the gut microbiome plays a critical role in the development of neurodegenerative and neurological diseases^16^. We have previously shown that in comparison to WT mice, the gut microbiota composition of *Cln2*^*R207X*^ mice is significantly altered^17^. Here we examined if acidified drinking water caused changes in the gut microbiota of *Cln2*^*R207X*^ mice, and if there were specific changes that correlated with the beneficial neurological effects of acidified water. The microbiota composition of fecal samples was determined by 16S rRNA gene sequencing. Alpha diversity, the microbial diversity within a sample, was quantified using the Chao 1 bias-corrected diversity index. Alpha diversity was similar in *Cln2*^*R207X*^ and WT mice receiving non-acidified drinking water, and acidified water did not cause statistically significant changes in either genotype (Fig. 4a). Alpha diversities in *Cln2*^*R207X*^ and WT mice receiving acidified drinking water, however, were close to being different with a Kruskal–Wallis p = 0.05 (Fig. 4a). The global gut microbiota compositions in the different groups (beta diversity) were compared by principal coordinate analysis. Beta diversity in *Cln2*^*R207X*^ mice was significantly different from that in WT mice on both types of drinking water (p = 0.00216 and 0.02814) (Fig. 4b). While acidified drinking water did not change the bacterial community structure in WT mice (p = 0.17749), it markedly altered the global microbiota composition in *Cln2*^*R207X*^ mice (p = 0.01082) (Fig. 4b).

Gut microbiota analysis at the individual taxonomic categories showed numerous differences (Supplementary Table 1, Figs. S3, S4). Acidified drinking water significantly increased the abundance of the *Bacteroidetes* and *Patescibacteria* phyla (2.2- and 3.5-fold) in *Cln2*^*R207X*^ mice, whereas in WT mice, it reduced the abundance of the *Tenericutes* phylum (− 11.2-fold). Acidified water in *Cln2*^*R207X*^ mice markedly altered the abundance of 3 orders (*Flavobacteriales*, *Betaproteobacteriales*, *Pasteurellales*), 13 families (*Atopobiaceae*, *Muribaculaceae* , *Rikenellaceae*, *Flavobacteriaceae*, *Uncultured bacterium-1*, *Enterococcaceae*, *Uncultured-1*, *Clostridiaceae 1*, *Burkholderiaceae*, *Nitrosomonadaceae*, *Pasteurellaceae*, *Anaeroplasmataceae*, *Ambiguous_taxa-4*), and 17 genera (*Coriobacteriaceae UCG-002*, *Parvibacter*, *Uncultured-3*, *Uncultured bacterium-3*, *Tetragenococcus*, *Clostridium sensu stricto 1*, *Tyzzerella*, *Eubacterium coprostanoligenes group*, *Ruminiclostridium 6*, *Ruminococcaceae UCG-005*, *{Unknown Genus} Erysipelotrichaceae*, *Dubosiella*, *Faecalibaculum*, *Uncultured-9*, *Parasutterella*, *Rodentibacter*, *Ambiguous_taxa-8*) (Supplementary Table 1). Acidified drinking water in WT mice significantly changed the abundance of 3 classes (*Saccharimonadia*, *Alphaproteobacteria*, *Mollicutes*), 2 orders (*Rickettsiales*, *Anaeroplasmatales*), 2 families (Saccharimonadaceae *Anaeroplasmataceae*), and 1 genus (*Anaeroplasma*) (Supplementary Table 1).

## Discussion

Our study demonstrates that acidified drinking water administered to *Cln2*^*R207X*^ mice from postnatal day 21 significantly improves motor function, restores muscle strength to the WT level and prevents tremors as measured at 3 months of age. Acidified drinking water also changed disease trajectory, delaying the death of *Cln2*^*R207X*^ mice by 3 weeks. The gut microbiota of *Cln2*^*R207X*^ and WT mice significantly differed and acidified drinking water caused pronounced changes in the gut microbiota composition of *Cln2*^*R207X*^ mice.

The complete prevention of tremors by acidified drinking water in *Cln2*^*R207X*^ mice was surprising. However, we have previously found that acidified drinking water can cause striking changes in serum metabolite levels in WT mice that included amino acids and their metabolites, fatty acids and their metabolites and many glycerophospholipids^15^. Similar acidified water-induced changes in neuroactive metabolite levels in *Cln2*^*R207X*^ mice may be responsible for the prevention of tremor development. For example, the serum level of palmitic amide, a primary fatty acid amide derived from palmitic acid, was increased 43.4-fold by acidified drinking water in WT mice^15^. Fatty acid amides compete with endocannabinoids for binding to the active site of fatty acid amide hydrolase and thus, increase the concentration of endocannabinoids by preventing their degradation^18^. Cannabinoids are known to reduce tremors associated with neurodegenerative diseases^19^.

Acidified drinking water in *Cln2*^*R207X*^ mice, in comparison to WT mice, changed several locomotor and behavioral parameters as measured in the force-plate actimeter: the area covered and total degrees of left and right turns were increased, whereas space utilization (spatial statistic) and bout of low mobility were decreased (see Figs. S1, S2). Although, the significance of these parameters is not clear, the changes in them may represent potentially negative effects of acidified water in *Cln2*^*R207X*^ mice.

Since *Cln2*^*R207X*^ mice die very early, starting around 4 months of age, our study focused on the effects of acidified drinking water on neurological functions and life span, which are more important outcome measures than the pathological changes in the brain (accumulation of lysosomal storage material, astrocytosis and microglial activation) common in mouse models of the different Batten disease forms. Most *Cln2*^*R207X*^ mice in our colony died suddenly: 1 day they were fine or just had tremors and the next day they were found dead. Examination of the organs of *Cln2*^*R207X*^ mice by a veterinary pathologist could not identify a definitive causes of death^5^. A recent study, however, showed a temporal relationship between seizure activity and death in *Cln2*^*R207X*^ mice^20^. The most likely cause of sudden death associated with neurodegeneration and brain dysfunction is respiratory arrest as observed in epileptic patients^21^ and in a mouse model of Leigh syndrome, a progressive encephalomyelopathy^22^. The underlying cause is the suppression/dysfunction of respiratory centers in the brainstem. While acidified drinking water in *Cln2*^*R207X*^ males improved motor function, restored muscle strength to the WT level and prevented tremors, it only delayed the death by 3 weeks: the median survival age was increased from 16 to 19 weeks. In *Cln2*^*R207X*^ females, acidified water increased the median survival age from 15 to 18 weeks (see Fig. 3). Despite the similar effects on median survival in *Cln2*^*R207X*^ males and females, acidified drinking water changed the survival curves of males and females differently. While the survival curves of *Cln2*^*R207X*^ males crossed each other at 21 weeks of age, the female survival curves did not cross each other. At 18 weeks of age, all *Cln2*^*R207X*^ females receiving non-acidified water were dead but 47% of *Cln2*^*R207X*^ females receiving acidified water were still alive, indicating a more pronounced effect of acidified water on the survival of *Cln2*^*R207X*^ females. Future studies will determine if the delayed death in *Cln2*^*R207X*^ females is associated with neurological improvements like in *Cln2*^*R207X*^ males.

We have previously tested the effects of acidified drinking water on neurological functions in *Cln3*^*−/−*^ and *Cln1*^*R151X*^ mice, models of juvenile CLN3 and infantile CLN1 diseases^6, 7^. While *Cln3*^*−/−*^ mice were on the 129S6/SvEv genetic background, *Cln1*^*R151X*^ mice were on the same mixed 129S6/SvEv;C57BL/6J background as *Cln2*^*R207X*^ mice. Acidified drinking water in *Cln3*^*−/−*^ mice had a temporary effect, improving the pole climbing ability at 3 months but not at 6 months of age^6^. In *Cln1*^*R151X*^ mice, however, acidified water prevented the motor impairment at both 3 and 6 months as measured in the pole climbing test^7^. Acidified drinking water had a similar effect on the pole climbing ability of *Cln2*^*R207X*^ mice; they could climb down the vertical pole as quickly as WT mice at 3 months of age (see Fig. 1). These results indicate that acidified drinking water is more beneficial for *Cln1* and *Cln2* mutations than for a *Cln3* deletion, and/or the genetic background has a strong influence on the disease-modifying effects of acidified water.

Accumulated evidence shows that an altered gut microbiota, by its secreted metabolites and via the vagus nerve, contributes to disease development and progression in neurological and neurodegenerative disorders^23^. We have previously demonstrated markedly changed gut microflora in *Cln3*^*−/−*^ and *Cln1*^*R151X*^ mice^6, 7^, and now confirmed that the gut microbiota is also altered in *Cln2*^*R207X*^ mice (see Fig. 4). While acidified drinking water did not change alpha diversity of the gut microbiota in *Cln3*^*−/−*^ and *Cln2*^*R207X*^ mice, it temporarily decreased alpha diversity in *Cln1*^*R151X*^ mice at 3 months of age^7^. Beta diversity, the global gut microbiota composition, was prominently changed by acidified water in both *Cln1*^*R151X*^ and *Cln2*^*R207X*^ mice, but still remained different from that in WT mice. In contrast, acidified drinking water did not alter beta diversity in *Cln3*^*−/−*^ mice^6^. At the individual taxonomic levels, acidified drinking water caused significant changes in *Cln3*^*−/−*^, *Cln1*^*R151X*^ and *Cln2*^*R207X*^ mice, but the changes were specific to each mouse model. For instance, while the abundance of the short-chain fatty acid-producing bacterial family, *Erysipelotrichaceae* was markedly increased by acidified water in the gut of *Cln1*^*R151X*^ mice, it was unchanged in *Cln2*^*R207X*^ mice. Similarly, while acidified water highly elevated the abundance of the *Eubacterium coprostanoligenes group* genus (105.6-fold) in *Cln2*^*R207X*^ mice, it did not change the abundance of this genus in *Cln1*^*R151X*^ mice.

The acidified drinking water-caused changes in the gut microbiota of *Cln2*^*R207X*^ mice might contribute to the improved neurological function and delayed death, although it cannot be assumed that these events are causally related. Acidified water caused an 8.6-fold increase in the abundance of the *Ruminococcaceae UCG-005* genus in *Cln2*^*R207X*^ mice (see Supplementary Table 1). Bacteria in the *Ruminococcaceae UCG-005* genus produce butyrate and other short-chain fatty acids^24^ that prevent neuroinflammation by inhibiting microglial activation and secretion of pro-inflammatory cytokines^25^. Short-chain fatty acids from gut bacteria also have beneficial effects on neuronal function by modulating the levels of neurotransmitters and neurotrophic factors^25^, and provide neuroprotection in mouse models of Parkinson’s disease, stroke and encephalopathy^26–29^. Increased plasma cholesterol levels have been associated with neuroinflammation and impaired brain function^30^. Gut bacteria in the *Eubacterium coprostanoligenes group* genus reduce the host’s cholesterol level by converting cholesterol to coprostanol^31^, and acidified drinking water in *Cln2*^*R207X*^ mice increased the abundance of the *Eubacterium coprostanoligenes group* genus by 105.6-fold (Supplementary Table 1). The *Tetragenococcus* genus contains five lactic acid-producing species with probiotic potential^32^, and the *Tetragenococcus halophilus* species has been shown to have beneficial immunomodulatory effects in vitro and in vivo^33^. Acidified water in *Cln2*^*R207X*^ mice increased the abundance of the *Tetragenococcus* genus by 10.9-fold (Supplementary Table 1). Another change that might contribute to the beneficial effects of acidified water was a pronounced 34.9-fold decrease in the abundance of the pathogenic *Clostridium sensu stricto 1* genus (Supplementary Table 1). Bacteria in the *Clostridium sensu stricto 1* genus cause intestinal inflammation^34, 35^.

In summary, our results show that acidified drinking water had clear beneficial effects in *Cln2*^*R207X*^ mice. It improved the pole climbing ability to the WT level, restored muscle strength to the WT level, prevented the development of tremors, and slightly delayed death. Our study emphasizes that drinking water is a major environmental factor that can alter neurological functions and pathology in rodent disease models.

### Supplementary Information

Supplementary Figures.

Supplementary Table 1.

## Data Availability

The 16S rRNA gene sequencing data generated and analyzed during the current study are available in the Sequence Read Archive (SRA) of NCBI (https://www.ncbi.nlm.nih.gov/sra), with accession number PRJNA984750.

## Abbreviations

OTUs: Operational taxonomic units
WT: Wild-type
