# Supplementary material for: Acidified drinking water improves motor function, prevents tremors and changes disease trajectory in Cln2R207X mice, a model of late infantile Batten disease
Source: Sci Rep. 2023 Nov 6;13:19229. doi: 10.1038/s41598-023-46283-w (PMC10628098; doi:10.1038/s41598-023-46283-w)
Supplement: Supplementary file 1 — Supplementary Figures. [file 41598_2023_46283_MOESM1_ESM.docx]

**Acidified drinking water improves motor function, prevents tremors and slightly delays death in *Cln2^R207X^* mice, a model of late infantile Batten disease**

Attila D. Kovács^1,2,^*, Jose L. Gonzalez Hernandez^3,4^, David A. Pearce^1,2^

^1^Pediatrics and Rare Diseases Group, Sanford Research, Sioux Falls, South Dakota, USA; ^2^Department of Pediatrics, Sanford School of Medicine, University of South Dakota, Sioux Falls, South Dakota, USA, ^3^Department of Agronomy, Horticulture, and Plant Science, and ^4^Department of Biology and Microbiology, South Dakota State University, Brookings, South Dakota, USA

***Corresponding author:**

Attila D. Kovács, PhD

Pediatric and Rare Diseases Group, Sanford Research

2301 E. 60^th^ Street N.,

Sioux Falls, South Dakota, 57014

Tel: +1 605-312-6404

E-mail: [Attila.Kovacs@sanfordhealth.org](mailto:Attila.Kovacs@sanfordhealth.org)

**Supplementary Figures**

**
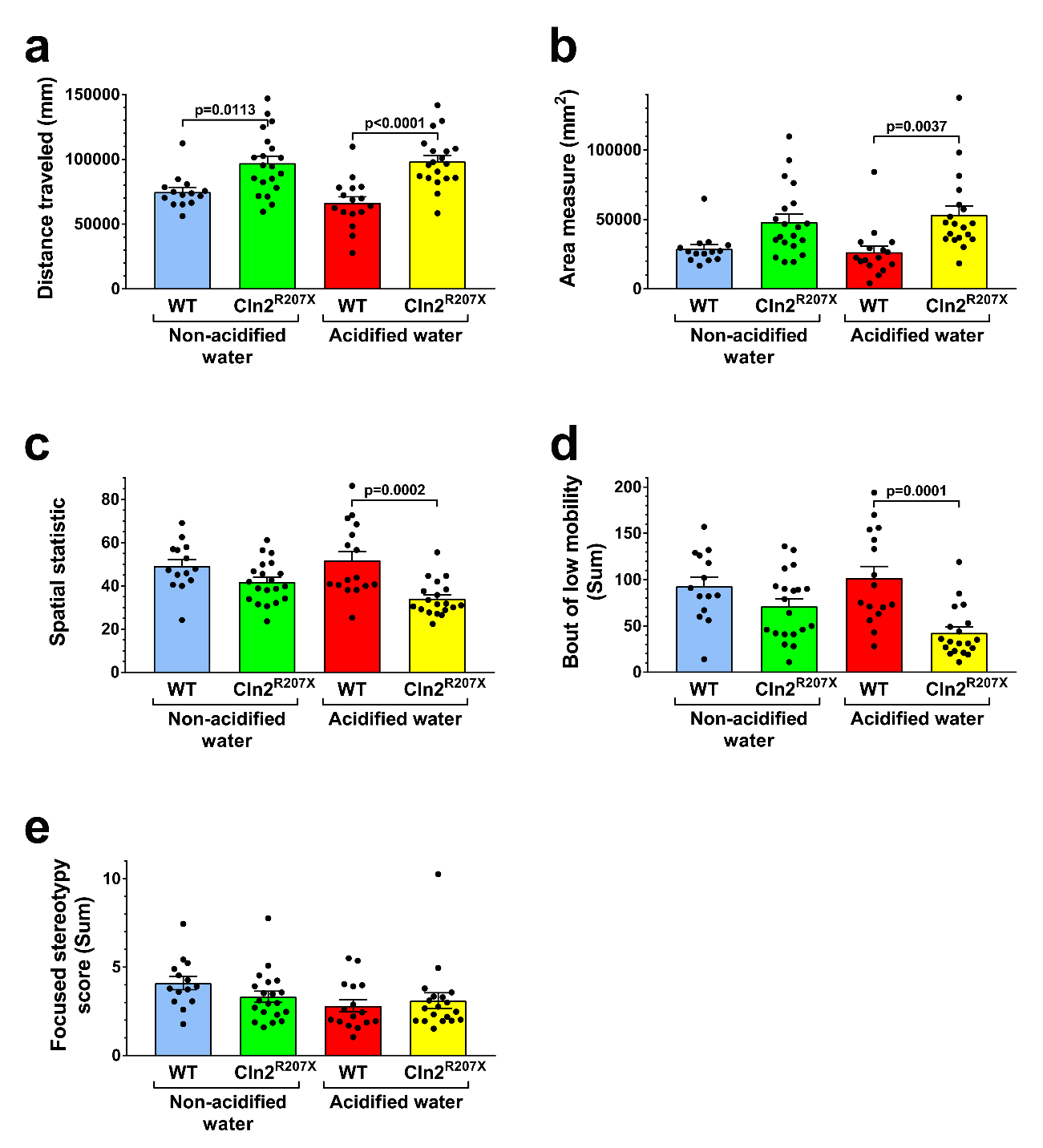
**

**Supplementary Figure 1. Effects of acidified drinking water on locomotor and behavioral parameters measured in a force-plate actimeter.** A group of *Cln2^R207X^* and wild-type (WT) male mice received acidified drinking water from weaning (21 days of age) and were compared to male mice that always had non-acidified drinking water. At the age of 3 months, mice were tested in a force-plate actimeter, which measures several behavioral parameters in freely moving animals. The force-plate actimeter recorded data for 10.24 minutes, in thirty 20.48-second frames, averaging 1,024 data points in each frame. **a)** Total distance traveled. **b)** Area covered. **c)** Spatial statistic (space utilization). **d)** Bout of low mobility. **e)** Focused stereotypes (head bobbing, grooming, rearing, scratching, etc.). Columns and bars represent mean + SEM and the symbols show the individual data (WT and *Cln2^R207X^* on non-acidified water: 14 and 20 mice; WT and *Cln2^R207X^* on acidified water: 16 and 19 mice). Statistical significance was determined by 2-way ANOVA with Tukey’s post-test for multiple comparisons.

**
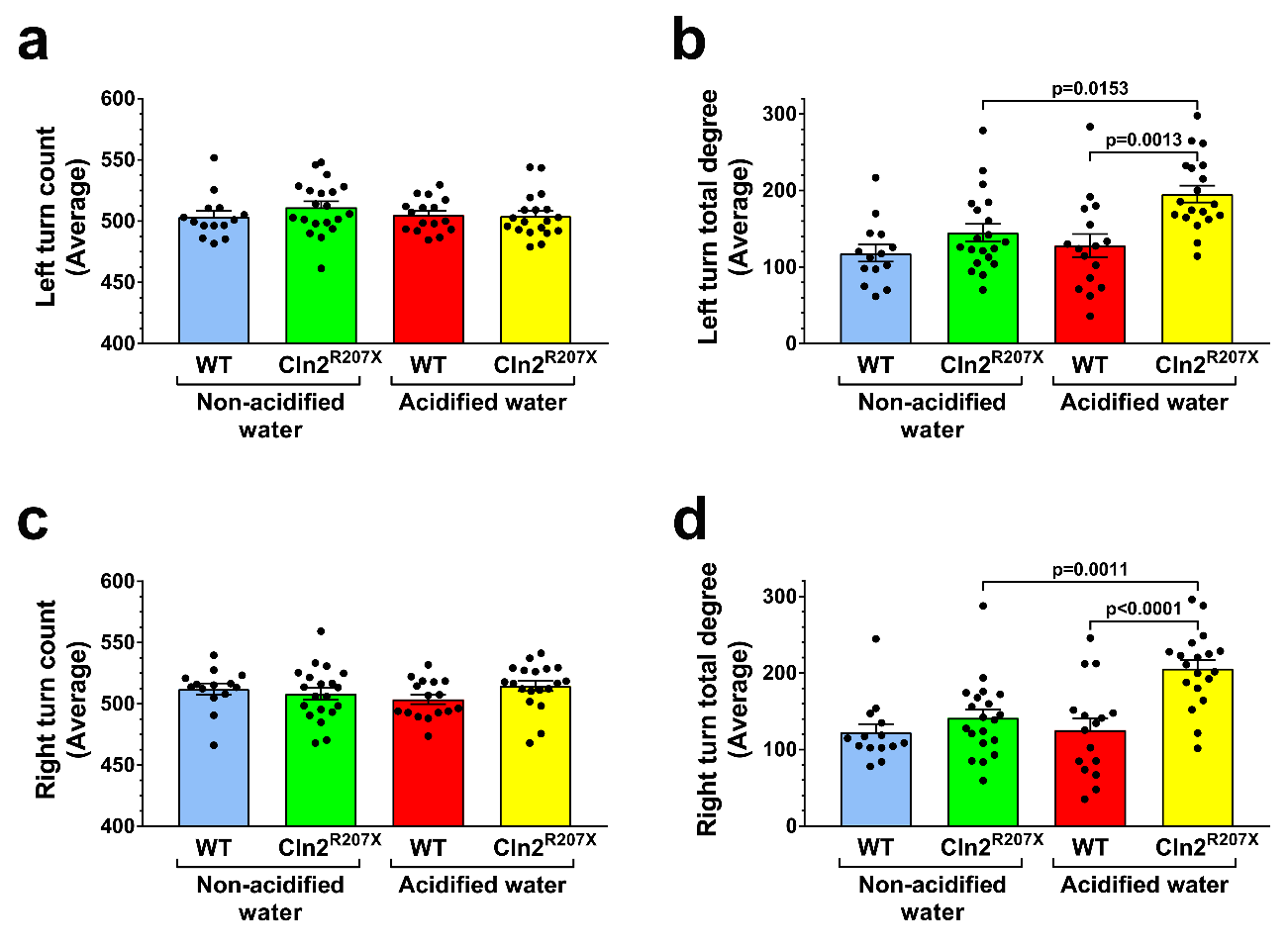
Supplementary Figure 2. Effects of acidified drinking water on additional locomotor parameters measured in a force-plate actimeter.** A group of *Cln2^R207X^* and wild-type (WT) male mice received acidified drinking water from weaning (21 days of age) and were compared to male mice that always had non-acidified drinking water. At the age of 3 months, mice were tested in a force-plate actimeter, which measures several behavioral parameters in freely moving animals. The force-plate actimeter recorded data for 10.24 minutes, in thirty 20.48-second frames, averaging 1,024 data points in each frame. **a)** Left turn count. **b)** Left turn total degree. **c)** Right turn count. **d)** Right turn total degree. Columns and bars represent mean + SEM and the symbols show the individual data (WT and *Cln2^R207X^* on non-acidified water: 14 and 20 mice; WT and *Cln2^R207X^* on acidified water: 16 and 19 mice). Statistical significance was determined by 2-way ANOVA with Tukey’s post-test for multiple comparisons.


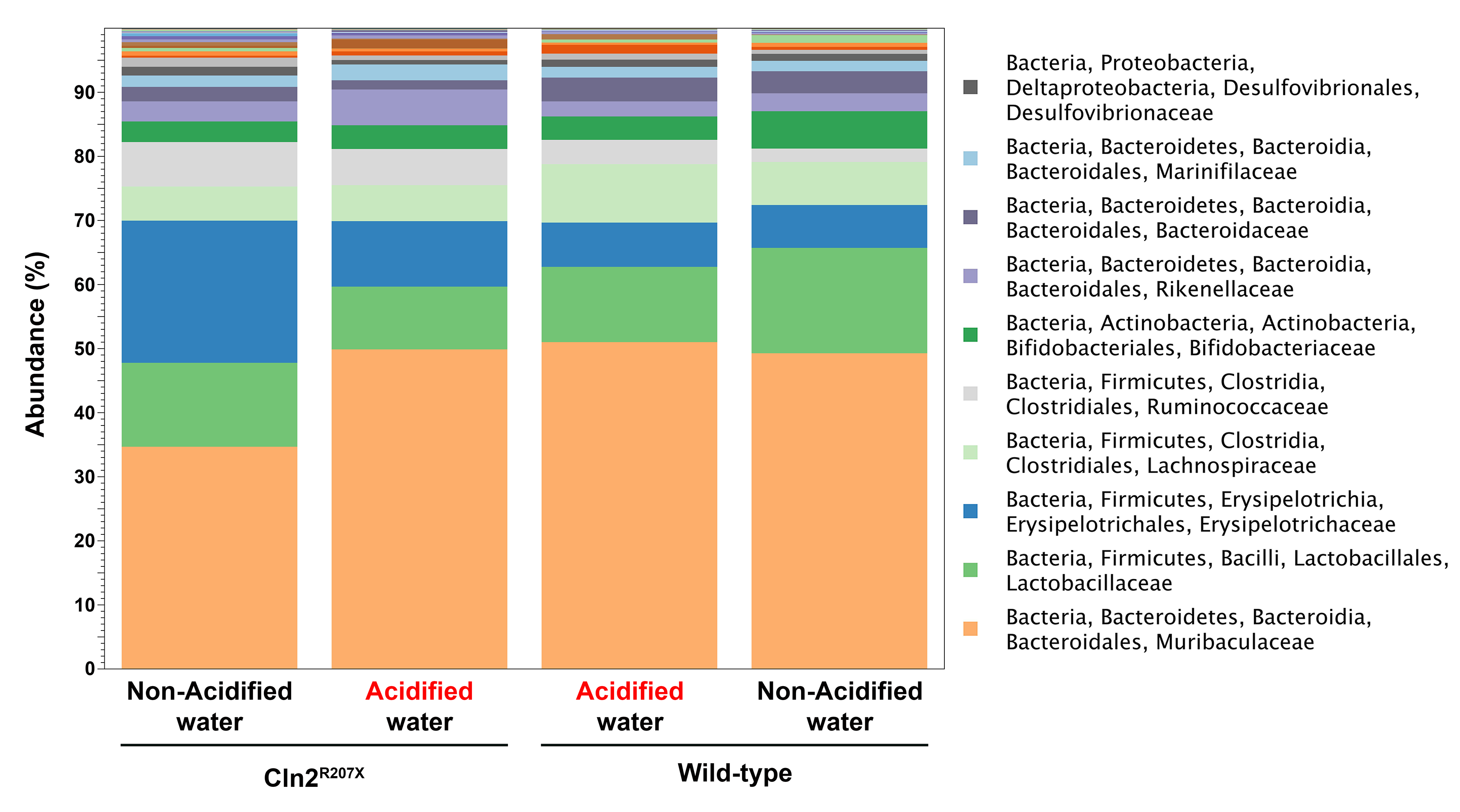


**Supplementary Figure 3. Representation of bacterial families in the gut microbiota of *Cln2^R207X^* and wild-type mice kept on non-acidified water or receiving acidified water from postnatal day 21.** A group of *Cln2^R207X^* and wild-type (WT) male mice received acidified drinking water from weaning (21 days of age) and were compared to male mice that always had non-acidified drinking water. Fecal pellets were collected at 3 months of age for the analysis of the gut microbiota by 16S rRNA gene sequencing. The stacked bar graph shows the percent composition of the gut microbiota at the family taxonomic level (averaged from 6 mice for each group).

**
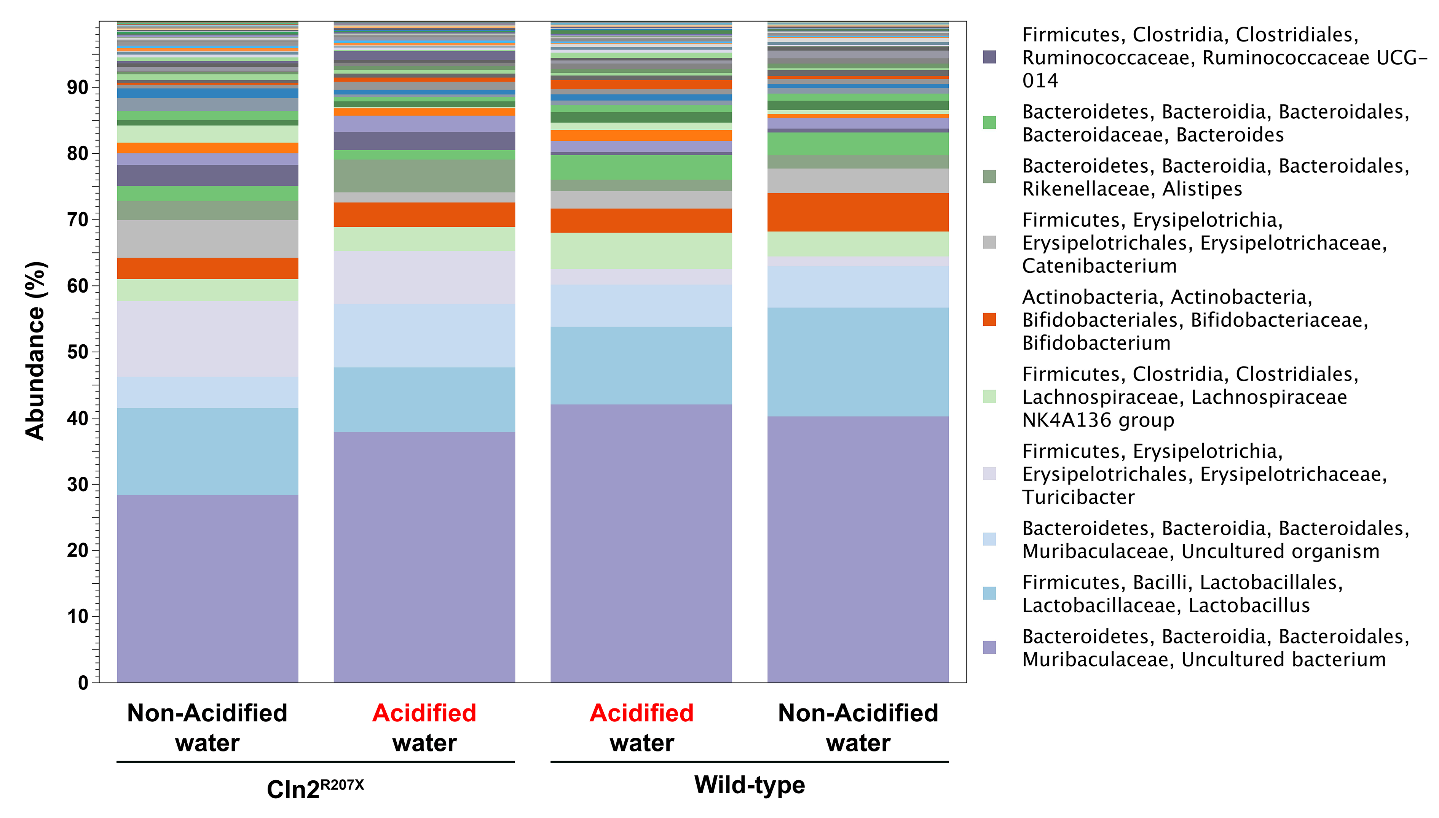
**

**Supplementary Figure 4. Representation of bacterial genera in the gut microbiota of *Cln2^R207X^* and wild-type mice kept on non-acidified water or receiving acidified water from postnatal day 21.** A group of *Cln2^R207X^* and wild-type (WT) male mice received acidified drinking water from weaning (21 days of age) and were compared to male mice that always had non-acidified drinking water. At 3 months of age, fecal pellets were collected for analysis of the gut microbiota by 16S rRNA gene sequencing. The stacked bar graph shows the percent composition of the gut microbiota at the genus taxonomic level (averaged from 6 mice for each group).
